# Supplementary material for: Phenothiazines Rapidly Induce Laccase Expression and Lignin-Degrading Properties in the White-Rot Fungus Phlebia radiata
Source: J Fungi (Basel). 2023 Mar 18;9(3):371. doi: 10.3390/jof9030371 (PMC10053029; doi:10.3390/jof9030371)
Supplement: Supplementary file 1 [file jof-09-00371-s001.zip › 2023_03_07_jof-2212467-supplementary.pdf]

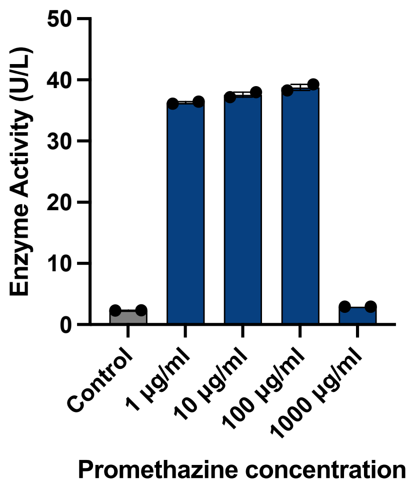

Supplementary Figure S1. Titration of promethazine concentrations and the induction of laccase activity in *P. radiata*. *P. radiata* mycelia were grown in liquid YNB + 2% glucose media and treated with different amounts of promethazine to identify concentrations that induce laccase activity. Control cells were not treated with promethazine. Laccase activity in the media supernatant was measured 3 days post-treatment using ABTS oxidation assays. Bars represent the mean of 2 biological replicates, with individual data points shown, and error bars represent standard error of the mean.

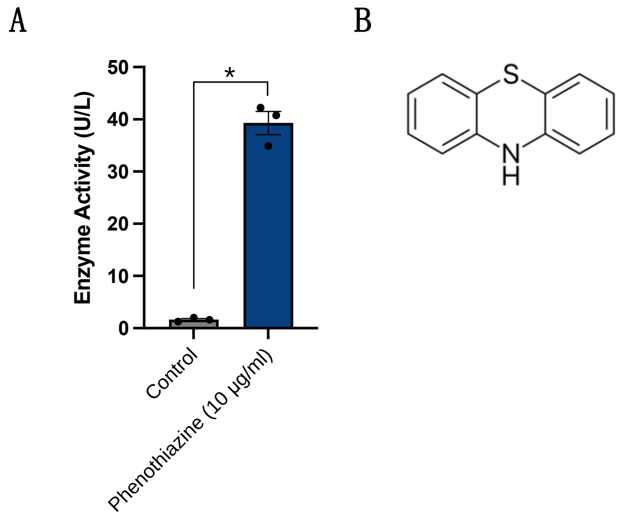

Supplementary Figure S2. Testing the ability of phenothiazine to induce laccase activity in *P. radiata.* The chemical phenotype microarray screen identified several phenothiazine derivatives as inducers of laccase activity in *P. radiata.* **(A)** Here, the phenothiazine backbone itself was tested for its ability to induce laccase activity. *P. radiata* was treated with phenothiazine and laccase activity was measured 2 days post-treatment using ABTS oxidation assays. Bars represent the mean of 3 biological replicates, with individual data points shown, and error bars represent standard error of the mean. Statistical significance is denoted by * for $P < 0.01$. **(B)** Chemical structure of phenothiazine for reference.

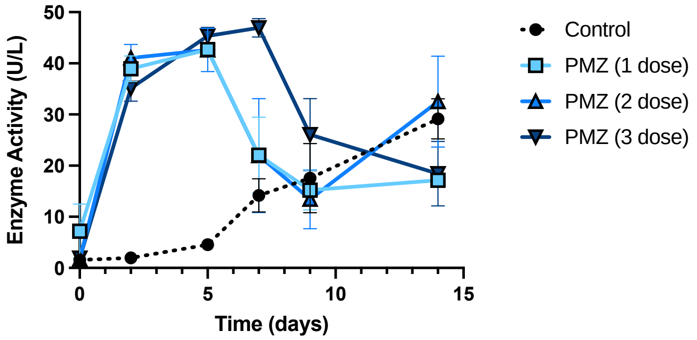

Supplementary Figure S3. Sequential doses of promethazine and prolonged laccase activity by *P. radiata*. Time-course studies of laccase induction by promethazine revealed a peak in laccase activity between 2-5 days post-treatment, followed by a decline in laccase activity. Here, we tested whether sequential exposures to promethazine could prolong laccase activity in media supernatants by treating *P. radiata* with either 1, 2 or 3 doses of promethazine during the time-course. *P. radiata* cultures receiving 1 dose were only exposed to promethazine on day 0. *P radiata* cultures receiving 2 doses received promethazine treatment on day 0 and day 2, and cultures receiving 3 doses received promethazine treatment on day 0, day 2 and day 5. Control cells were not exposed to promethazine. Data points represent the mean of three biological replicates and error bars represent the standard deviation.

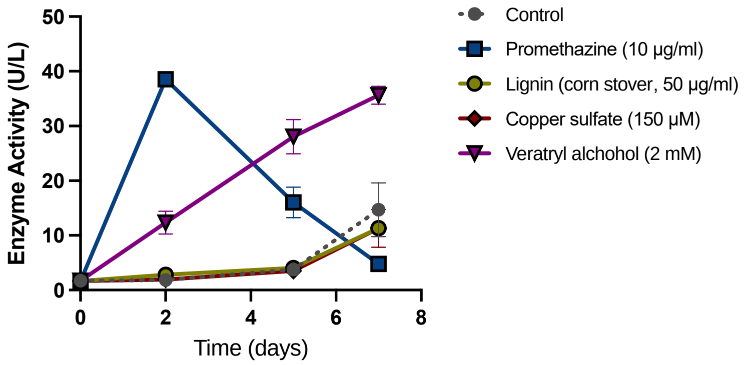

Supplementary Figure S4. Time-course analysis of laccase induction resulting from promethazine treatment or other known laccase-inducing compounds. Laccase activity assays were performed on media samples collected from 20 ml *P. radiata* cultures grown in YNB + 2% glucose at multiple timepoints post-promethazine treatment. For all induction tests, the final induction solution represented 0.1% of the total culture volume. Data points represent the mean of n=3 biological replicates and error bars represent the standard error of the mean.

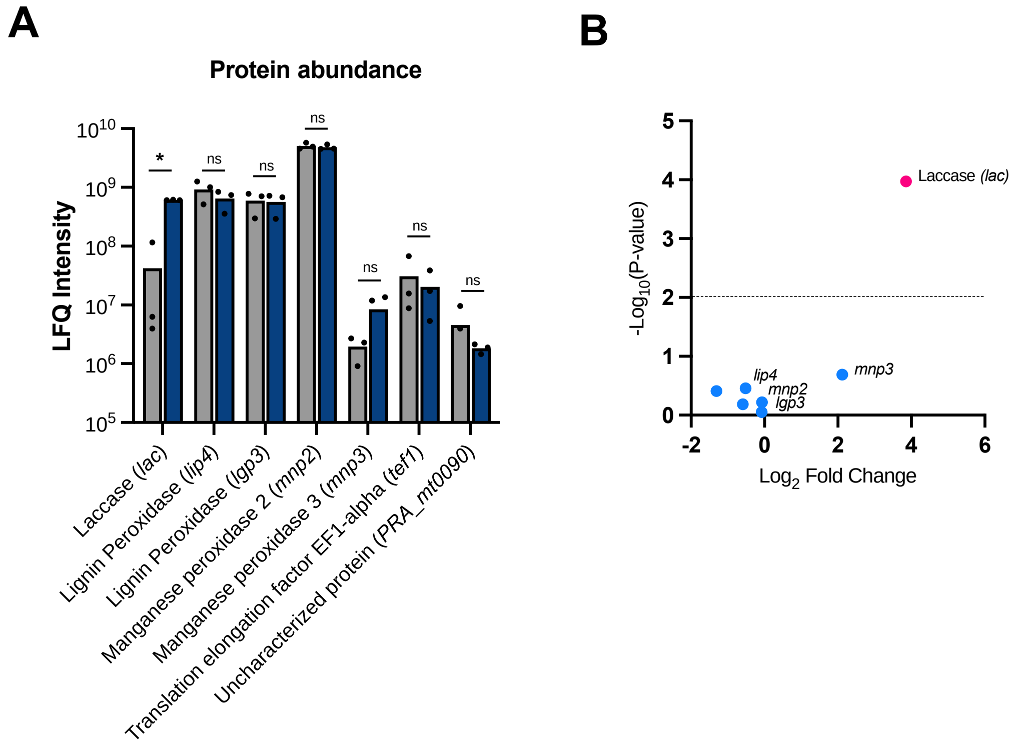

Supplementary Figure S5. Proteomics analyses of *P. radiata* secreted proteins in response to promethazine-treatment. (A) The abundance of proteins identified in *P. radiata* conditioned media were measured at 2-days post-promethazine treatment using label-free quantitative proteomics analysis. Bars represent the mean of n=3 biological replicates with individual data points shown (* denotes $P < 0.01$, and "ns" denotes not significant). **(B)** Volcano plot depicting differential protein expression analysis of promethazine-treated vs. non-treated controls. Only proteins with known functions in wood-degradation are labeled on the plot for clarity. Red dots represent statistically significant differential protein expression (fold-change > 2 and $P < 0.01$), and blue dots represent not statistically significant. The dotted line is added for reference of $P = 0.01$.

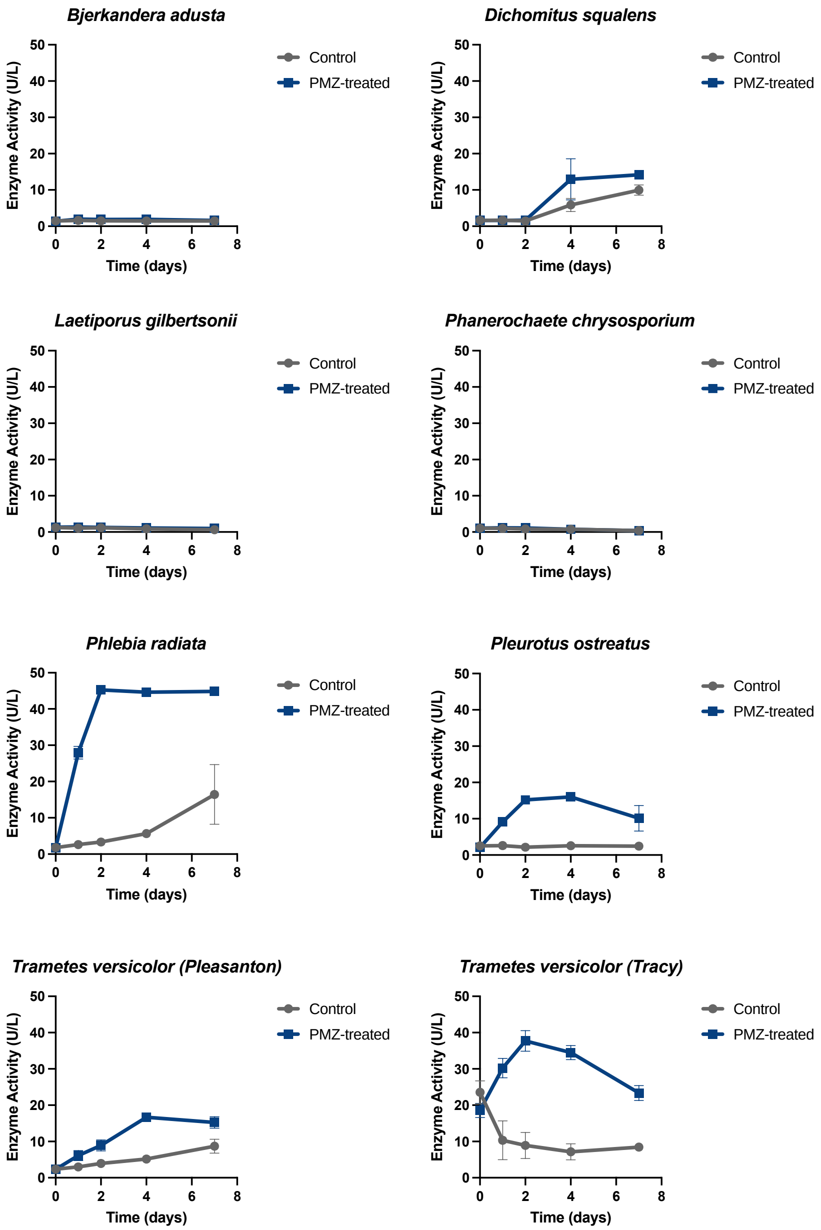

Supplementary Figure S6. Multi-species time-course examination of laccase activity in response to promethazine treatment. Laccase activity in media supernatants were measured from diverse fungal species +/- promethazine treatment. Samples were analyzed for extracellular laccase activity at 0, 1, 2, 4 and 7-days post-treatment using ABTS oxidation assays. Data points represent the mean of n = 3 biological replicates and error bars depict the standard error of the mean.

PM Plate	Wells	Chemical
PM21D	A 01 - 04	Guanidine hydrochloride
PM21D	A 05 - 08	2,2`-Dipyridyl
PM21D	A 09 - 12	Promethazine
PM21D	B 01 - 04	Nystatin
PM21D	B 05 - 08	Dodecyltrimethyl ammonium bromide
PM21D	B 09 - 12	Protamine sulfate
PM21D	C 01 - 04	Cetylpyridinium chloride
PM21D	C 05 - 08	Domiphen bromide
PM21D	C 09 - 12	L-Aspartic acid b-hydroxamate
PM21D	D 01 - 04	Pyrithione
PM21D	D 05 - 08	EDTA
PM21D	D 09 - 12	Sodium dichromate
PM21D	E 01 - 04	Compound 48/80
PM21D	E 05 - 08	Manganese(II) chloride
PM21D	E 09 - 12	Magnesium chloride
PM21D	F 01 - 04	Copper(II) sulfate
PM21D	F 05 - 08	Neomycin
PM21D	F 09 - 12	D-Cycloserine
PM21D	G 01 - 04	Sodium selenite
PM21D	G 05 - 08	Nickel(II) chloride
PM21D	G 09 - 12	Trifluoperazine
PM21D	H 01 - 04	Diamide
PM21D	H 05 - 08	Thiourea
PM21D	H 09 - 12	Zinc chloride
PM22D	A 01 - 04	L-Glutamic acid g-monohydroxamate
PM22D	A 05 - 08	Sodium Metavanadate
PM22D	A 09 - 12	Caffeine
PM22D	B 01 - 04	L-Arginine hydroxamate
PM22D	B 05 - 08	Glycine hydroxamate
PM22D	B 09 - 12	Triclosan
PM22D	C 01 - 04	3-Amino-1,2,4-triazole
PM22D	C 05 - 08	Miltefosine
PM22D	C 09 - 12	DL-Serine hydroxamate
PM22D	D 01 - 04	Polymyxin B
PM22D	D 05 - 08	Urea hydrogen peroxide
PM22D	D 09 - 12	Sodium arsenate
PM22D	E 01 - 04	CCCP

PM22D	E 05 - 08	BAPTA
PM22D	E 09 - 12	D-Serine
PM22D	F 01 - 04	Azaserine
PM22D	F 05 - 08	Lithium chloride
PM22D	F 09 - 12	FCCP
PM22D	G 01 - 04	Benzamidine hydrochloride
PM22D	G 05 - 08	Cycloheximide
PM22D	G 09 - 12	Thallium(I) acetate
PM22D	H 01 - 04	Bleomycin
PM22D	H 05 - 08	Paromomycin
PM22D	H 09 - 12	Myclobutanil
PM23A	A 01 - 04	Benzethonium chloride
PM23A	A 05 - 08	Chlorpromazine
PM23A	A 09 - 12	Ammonium sulfate
PM23A	B 01 - 04	Cadmium chloride
PM23A	B 05 - 08	Dequalinium chloride
PM23A	B 09 - 12	Doxycycline
PM23A	C 01 - 04	Glycine hydrochloride
PM23A	C 05 - 08	Hydroxylamine
PM23A	C 09 - 12	Poly-L-lysine
PM23A	D 01 - 04	Chromium(III) chloride
PM23A	D 05 - 08	Cobalt(II) chloride
PM23A	D 09 - 12	Cupric(II) chloride
PM23A	E 01 - 04	Sodium metaborate
PM23A	E 05 - 08	Sodium metaperiodate
PM23A	E 09 - 12	Sodium metaarsenite
PM23A	F 01 - 04	Sodium azide
PM23A	F 05 - 08	Caprylic acid
PM23A	F 09 - 12	Sodium cyanate
PM23A	G 01 - 04	Sodium nitrite
PM23A	G 05 - 08	Sodium orthovanadate
PM23A	G 09 - 12	2-Deoxy-D-glucose
PM23A	H 01 - 04	Sodium selenate
PM23A	H 05 - 08	Sodium cyanide
PM23A	H 09 - 12	Sodium thiosulfate
PM24C	A 01 - 04	Apramycin
PM24C	A 05 - 08	9-Aminoacridine
PM24C	A 09 - 12	Zaragozic acid A
PM24C	B 01 - 04	Blasticidin S

PM24C	B 05 - 08	Thioridazine
PM24C	B 09 - 12	Sodium benzoate
PM24C	C 01 - 04	Chlortetracycline
PM24C	C 05 - 08	Sodium metasilicate
PM24C	C 09 - 12	Pentamidine isethionate
PM24C	D 01 - 04	6-Azauracil
PM24C	D 05 - 08	Potassium chromate
PM24C	D 09 - 12	Thialysine
PM24C	E 01 - 04	Berberine chloride
PM24C	E 05 - 08	EGTA
PM24C	E 09 - 12	Sodium pyrophosphate
PM24C	F 01 - 04	Isoniazid
PM24C	F 05 - 08	Methyl viologen
PM24C	F 09 - 12	Sodium fluoride
PM24C	G 01 - 04	Cisplatin
PM24C	G 05 - 08	Aluminum sulfate
PM24C	G 09 - 12	Fluconazole
PM24C	H 01 - 04	Propiconazole
PM24C	H 05 - 08	Tamoxifen
PM24C	H 09 - 12	Miconazole
PM25D	A 01 - 04	Hydroxyurea
PM25D	A 05 - 08	Tobramycin
PM25D	A 09 - 12	Niaproof
PM25D	B 01 - 04	b-Chloro-L-alanine
PM25D	B 05 - 08	Tetrazolium Violet
PM25D	B 09 - 12	Kanamycin
PM25D	C 01 - 04	4-Aminopyridine
PM25D	C 05 - 08	Amitriptyline
PM25D	C 09 - 12	4-Nitroquinoline N-oxide
PM25D	D 01 - 04	Alexidine
PM25D	D 05 - 08	Hygromycin B
PM25D	D 09 - 12	5-Fluorodeoxyuridine
PM25D	E 01 - 04	Sodium salicylate
PM25D	E 05 - 08	Succinic acid
PM25D	E 09 - 12	Clomiphene
PM25D	F 01 - 04	DL-Malic acid
PM25D	F 05 - 08	Tartaric acid
PM25D	F 09 - 12	Fumaric acid
PM25D	G 01 - 04	5-Fluorocytosine

PM25D	G 05 - 08	Palladium(II) chloride
PM25D	G 09 - 12	Ibuprofen
PM25D	H 01 - 04	Chloroquine
PM25D	H 05 - 08	trans-Cinnamic acid
PM25D	H 09 - 12	5-Fluorouracil

Supplementary Table S1. Chemicals used in Phenotype Microarray Experiments and their locations in PM plates. List of compounds tested using Biolog chemical sensitivity tests for fungi phenotype microarrays. Each chemical was tested across a range of 4 concentrations (exact concentrations are proprietary information of Biolog Inc.)
